# Identification, Molecular Cloning and Expression Analysis of Five RNA-Dependent RNA Polymerase Genes in *Salvia miltiorrhiza*

**DOI:** 10.1371/journal.pone.0095117

**Published:** 2014-04-14

**Authors:** Fenjuan Shao, Shanfa Lu

**Affiliations:** Institute of Medicinal Plant Development, Chinese Academy of Medical Sciences & Peking Union Medical College, Beijing, China; East Carolina University, United States of America

## Abstract

RNA-dependent RNA polymerases (RDRs) act as key components of the small RNA biogenesis pathways and play significant roles in post-transcriptional gene silencing (PTGS) and antiviral defense. However, there is no information about the *RDR* gene family in *Salvia miltiorrhiza*, an emerging model medicinal plant with great economic value. Through genome-wide predication and subsequent molecular cloning, five full-length *S. miltiorrhiza RDR* genes, termed *SmRDR1*–*SmRDR5*, were identified. The length of *SmRDR* cDNAs varies between 3,262 (*SmRDR5*) and 4,130 bp (*SmRDR3*). The intron number of *SmRDR* genes varies from 3 (*SmRDR1, SmRDR3* and *SmRDR4*) to 17 (*SmRDR5*). All of the deduced SmRDR protein sequences contain the conserved RdRp domain. Moreover, SmRDR2 and SmRDR4 have an additional RRM domain. Based on the phylogenetic tree constructed with sixteen RDRs from *Arabidopsis*, rice and *S. miltiorrhiza*, plant RDRs may be divided into four groups (RDR1–RDR4). The RDR1 group contains an AtRDR and an OsRDR, while includes two SmRDRs. On the contrary, the RDR3 group contains three AtRDRs and two OsRDRs, but has only one SmRDR. *SmRDRs* were differentially expressed in flowers, leaves, stems and roots of *S. miltiorrhiza* and responsive to methyl jasmonate treatment and cucumber mosaic virus infection. The results suggest the involvement of *RDRs* in *S. miltiorrhiza* development and response to abiotic and biotic stresses. It provides a foundation for further studying the regulation and biological functions of *SmRDRs* and the biogenesis pathways of small RNAs in *S. miltiorrhiza*.

## Introduction

RNA-dependent RNA polymerases (RDRs), which catalyze the conversion of single-stranded RNAs (ssRNAs) into double-stranded ones, play vital roles in the production of various small interfering RNA (siRNA) species in plants through collaboration with other proteins, such as Dicer-likes (DCLs) capable of cleaving double-stranded RNAs (dsRNAs) into 21–24 nt duplexes [1], [2]. Based on the origins and biogenesis pathways, siRNAs generated from dsRNAs can be classified into several groups, such as trans-acting small interfering RNAs (ta-siRNAs), heterochromatic siRNAs (hsiRNAs) and natural antisense transcript-derived siRNAs (nat-siRNAs) [3]–[5]. These siRNAs may incorporated into the Argonaute (AGO)-containing RNA-induced silencing complexes (RISCs) to silence a variety of gene transcripts, repetitive sequences, sense transgenes, viruses and mobile elements through RNA cleavage, translational inhibition, DNA methylation and heterochromatin formation [6].

RDR proteins are characterized by the conserved RNA-dependent RNA polymerase catalytic domain (RdRp) and are among the first components identified for plant small RNA biogenesis pathways [7]–[10]. They are present in fungi, viruses, plants and nematodes, but have not been found in insects and vertebrates [11], [12]. The activity of RDR was detected in Chinese cabbage more than forty years ago [8], whereas the cDNA of *RDR* was first isolated from tomato in 1998 [13]. So far, *RDR* genes have been identified in various plant species, such as *Arabidopsis thaliana*, rice, maize, tomato and tobacco [14]–[17]. Similar to *DCLs* and *AGOs*, *RDR* genes exist as a family in plants and the number of *RDR* genes may be not the same in different species. For instance, there are six *RDR* members in *Arabidopsis* and *Solanum lycopersicum* [14], five in rice [15] and maize [16], and at least three in *Nicotiana tobaccum* and *N. attenuate* [17]. Each small RNA biogenesis pathway may involve different member of the RDR family.

The six members included in the *A. thaliana AtRDR* gene family were termed *AtRDR1*–*AtRDR6*, respectively [14]. *AtRDR1*, *AtRDR2* and *AtRDR6* play distinct and overlapping functions in various aspects, such as viral resistance, chromatin silencing and post-transcriptional gene silencing (PTGS) [19], [20]. *AtRDR1* and its ortholog in tobacco, *NtRDR1*, are induced by salicylic acid (SA) treatment and virus infection and involved in plant susceptibility to tobacco mosaic tobamovirus (TMV) and tobacco rattle virus (TRV) [21]. The underlying functional mechanism of *AtRDR1* appears to produce and amplify exogenous and virus-derived siRNAs (vsiRNAs) in infected plants [19]–[24]. In addition to antiviral responses, *RDR1* plays a significant role in plant resistance to herbivore attack [18]. *AtRDR2* is involved in the production of the most abundant endogenous hsiRNAs that are mostly 24 nt in length and are associated with heterochromatic and repetitive regions, such as the pericentromeric regions and telomeres [25]. RDR2-dependent RNA-directed DNA methylation (RdDM) is responsible for siRNA-mediated DNA methylation and histone modifications at *Arabidopsis* telomeres, and is required for the maintenance of telomeric heterochromatin [25]. In addition, *AtRDR2* is involved in the development of the female gametophyte [26]. *AtRDR6*, acts in various gene silencing pathways and plays important roles in the biogenesis of ta-siRNA and nat-siRNA. It is also well-known for the amplification of improper terminated and unpolyadenylated RNAs generated from transgenes or inverted repeats to trigger degradation of complementary RNA species [27]. The functions of *AtRDR3*–*AtRDR5* are currently unknown [7].

Although *RDR* genes have been isolated from various plant species, to our best knowledge, there is no report on *RDRs* in medicinal plants. *Salvia miltiorrhiza*, well-known as danshen in Chinese, is an economically significant medicinal plant and is an emerging model medicinal plant for Traditional Chinese Medicine (TCM) studies [28]. It has been used for treating various human diseases, such as dysmenorrhoea, amenorrhoea and cardiovascular disease, for thousands of years [28]–[30]. The *S. miltiorrhiza* genome has been preliminarily decoded (Chen et al., unpublished data). We have previously analyzed the *S. miltiorrhiza AGO* gene families involved in small RNA production and action [31]. In order to characterize the *RDR* genes in *S. miltiorrhiza*, we performed a genome-wide search of *RDRs* against the working draft of the *S. miltiorrhiza* genome, followed by molecular cloning and molecular analysis. The results will be a basis for understanding the gene silencing pathways in *S. miltiorrhiza*.

## Materials and Methods

### Plant materials and stress treatment

*S. miltiorrhiza* Bunge (line 993) were grown in a field nursery. Flowers, leaves, stems and roots were collected from two-year-old plants and stored in liquid nitrogen until use. Plantlets cultivated *in vitro* were grown at 25°C with a photoperiod of 16 h light and 8 h dark for six weeks as described previously [32]. MeJA treatment were carried out following the procedures reported previously [32], [33]. Plantlets were treated for 12, 24, 36 and 48 h and then sampled. Sterile water-treated plantlets were used as controls. For cucumber mosaic virus (CMV) infection, the silicon carbide powder friction method was used. Briefly, leaves of six-week-old plantlets cultivated *in vitro* were dusted with silicon carbide powder and then inoculated with CMV subgroup I for 12, 24, 48 and 72 h. Leaves inoculated with phosphate buffered saline (PBS) were used as controls. All tissues collected were stored in liquid nitrogen until use. Three independent biological replicates were performed for each experiment.

### Identification of *SmRDR* genes

*Arabidopsis* and rice RDR protein sequences were downloaded from GenBank (http://www.ncbi.nlm.nih.gov/protein). It includes AtRDR1 (AEE29226.1), AtRDR2 (AEE82976.1), AtRDR3 (O82190.2), AtRDR4 (O82189.2), AtRDR5 (O82188.2), AtRDR6 (AEE78550.1), OsRDR1 (Q0DXS3.2), OsRDR2 (Q7XM31.1), OsRDR3 (Q5QMN5.2), OsRDR4 (Q5QMN4.2), and OsRDR6 (Q8LHH9.1). *S. miltiorrhiza* SmRDR genes were predicted by BLAST analysis of *Arabidopsis* and rice RDRs against the working draft of the *S. miltiorrhiza* genome (Chen et al., unpublished data) using tBLASTn [34], [35]. The retrieved genomic DNA sequences were used for gene model prediction on the GENSCAN web server (http://genes.mit.edu/GENSCAN.html). Gene models were manually corrected according to the alignment between SmRDRs and other plant RDRs obtained from BLAST analysis of predicted SmRDRs against the non-redundant protein sequence (nr) database using the BLASTx algorithm (http://www.ncbi.nlm.nih.gov/BLAST).

### Molecular cloning of *SmRDR* cDNAs

The 5′-RACE and 3′-RACE were carried out as described previously [31]. Briefly, total RNA extracted from the root of *S. miltiorrhiza* was purified using the oligotex mRNA mini kit (Invitrogen). 5′ and 3′ RACE was performed on mRNA using the GeneRacer kit (Invitrogen). Gene specific nesting and nested primers were designed and synthesized (Tables S1 and S2). PCR products were purified, cloned and sequenced. Based on the obtained 5′ and 3′ cDNA sequence, gene-specific forward and reverse primers were designed and synthesized (Table S3). Full-length *SmRDR* cDNAs were PCR-amplified, cloned and sequenced as described [31].

### Bioinformatic analysis and phylogenetic tree construction

Bioinformatic analysis of SmRDR sequence features, such as intron/exon structures, molecular weight (MW), theoretical isoelectric point (pI) and conserved domain, were performed as described previously [31], [36]. The conserved motifs of SmRDRs were analyzed using Multiple Expectation Maximization for Motif Elicitation (MEME) version 4.9.1 [37] with the following parameters. Optimum motif width was set to≧6 and ≦50. The maximum number of motifs was designated to identify 20 motifs following the previous reported studies [17]. The conserved residues were analyzed by alignment of amino acid sequences using T-coffee [38]. Phylogenetic tree was constructed for protein sequences of sixteen RDRs from *S. miltiorrhiza*, *Arabidopsis* and rice using MEGA version 4.0 by the neighbor-joining method with 1,000 bootstrap replicates [39], [40].

### Quantitative real-time reverse transcription-PCR (qRT-PCR)

Expression of SmRDRs in roots, stems, leaves and flowers of 2-year-old *S. miltiorrhiza* plants and in plantlets treated with MeJA and CMV was analyzed using the qRT-PCR method as described previously [31]. Briefly, Gene-specific forward and reverse primers were designed and synthesized (Table S4). About 10 ng cDNA reversely transcribed from total RNA was used as a template in a 20 µl volume. *SmUBQ10* was used as a reference [31]. qPCR was carried out in triplicates for each biological sample using the BIO-RAD CFX system (Bio-Rad). Three fully independent biological replicates were performed. The specificity of amplification was assessed by dissociation curve analysis. Gene expression levels were determined using the 2^−ΔΔCq^ method, where Cq represents the threshold cycle [41]. Relative amount of transcripts was calculated and normalized as described previously [42]. Average Cq were log transformed, mean centered and autoscaled [42]. Standard deviations of mean value from three biological replicates were calculated as described previously [42].

## Results

### Identification and molecular cloning of five *S. miltiorrhiza RDR* genes

BLAST analysis of *Arabidopsis* and rice RDRs against the working draft of the *S. miltiorrhiza* genome (Chen et al., unpublished data) using tBLASTn [34], [35] showed the existence of five *SmRDR* gene loci in the *S. miltiorrhiza* genome. Genomic DNA sequences were retrieved and predicted for gene models on the GENSCAN web server (http://genes.mit.edu/GENSCAN.html). The five gene models computationally predicted were BLAST-analyzed against the non-redundant protein sequence (nr) database (http://www.ncbi.nlm.nih.gov/BLAST) using BLASTx with default parameters and then manually corrected according to the alignment between SmRDRs and other plant RDRs. To further experimentally validate the predicted cDNA sequences of *SmRDRs*, molecular cloning of full-length *SmRDR* cDNA was carried out using RNA ligase-mediated rapid amplification of 5′ (5′ RACE) and 3′ (3′ RACE) cDNA ends and subsequent PCR amplification of coding regions. The deduced amino acid sequences of all five SmRDRs share high sequence identity with known plant RDRs and contain the conserved RdRp domain, suggesting they are authentic SmRDRs. The identifed *SmRDR* genes are termed *SmRDR1*–*SmRDR5*, respectively. The cloned cDNAs have been submitted to GenBank under the accession numbers KF872203–KF872207.

### Gene structure and conserved domain analyses

**Figure 1:**
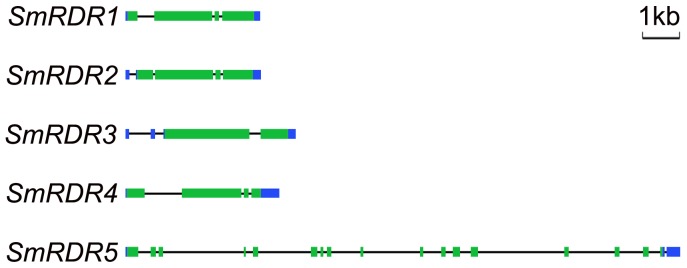
Gene structures of *S. miltiorrhiza SmRDRs*. Filled boxes represent exons with coding regions in green and 5′- and 3′-UTRs in blue. The connecting lines represent introns.

Sequence feature analysis of *SmRDRs* suggests that the length of open reading frames (ORFs) of SmRDRs varies from 2,697 (*SmRDR5*) to 3,588 bp (*SmRDR3*) (Table 1). The length of 5′ and 3′ UTRs varies between 35 and 293 bp and between 155 and 583 bp, respectively. The size of deduced *SmRDR* proteins varies between 898 and 1195 amino acids. The molecular weight (Mw) varies from 102.53 to 136.33 kDa, and the theorectical p*I* is between 6.92 and 8.22 (Table 1). The *SmRDR5* locus, which produces the shortest *SmRDR*, has seventeen introns (Fig. 1, Table 1). *SmRDR2* contains four introns including one is located within the 5′ untranslated region (UTR) (Fig. 1, Table 1). The rest three, including *SmRDR1*, *SmRDR3* and *SmRDR4*, have 3 introns (Fig. 1, Table 1). All of the introns of *SmRDR1* and *SmRDR4* are located in the coding regions; however, of the 3 introns of *SmRDR3*, only one is located in the coding region. The other two are located within the 5′ UTR. These 5′ UTR-located introns might enhance gene transcription and RNA stability [43]–[45]. Additionally, the size of introns varies significantly among *SmRDRs* (Fig. 1). It suggests the diversity of *SmRDRs* in sequence features and gene structures.

**Table 1:** Sequence features and intron number of *SmRDRs*.

Gene name	Accession number	cDNA (bp)	ORF (bp)	5′UTR (bp)	3′UTR (bp)	Protein (aa)	Mw (kDa)	p*I*	Intron no.
									
*SmRDR1*	KF872203	3606	3357	57	192	1118	127.93	8.22	3
*SmRDR2*	KF872204	3609	3306	148	155	1101	125.55	7.58	4
*SmRDR3*	KF872205	4130	3588	293	249	1195	136.33	7.67	3
*SmRDR4*	KF872206	3537	2919	35	583	972	110.04	6.92	3
*SmRDR5*	KF872207	3262	2697	65	500	898	102.53	7.07	17

**Figure 2:**
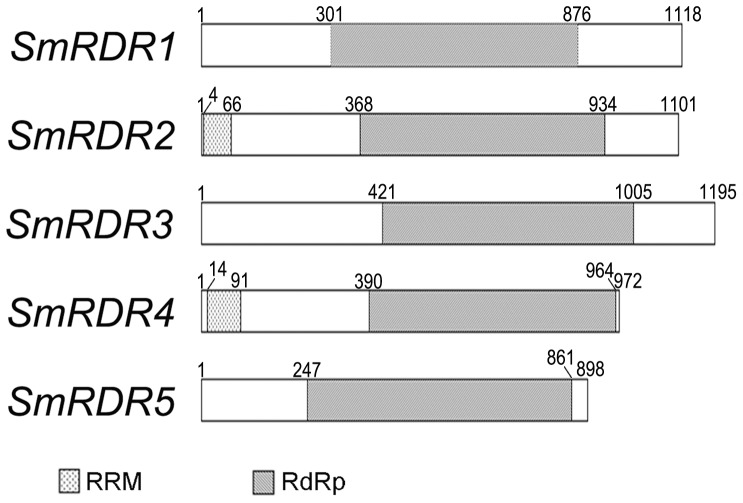
Conserved domains in SmRDR proteins. Boxes with points represent the RRM domain. Grey boxes represent the RdRp domain. The first and the last amino acids of SmRDR proteins and the location of RRM and RdRp domains are numbered.

The search for conserved domains in SmRDR proteins against the NCBI Conserved Domain Database showed that all of the five SmRDRs contained the conserved RdRp domain (Fig. 2). It is consistent with the results from other plant RDRs [15]–[17]. SmRDR2 and SmRDR4 have an additional RNA recognition motif (RRM) in the region close to the N-terminus (Fig. 2). RRM in SmRDR2 starts from the 4^th^ amino acid and ends at the 66^th^, while SmRDR4 RRM starts from the 14^th^ amino acid and ends at the 91^st^. The actual function of additional RRM in RDR proteins remains to be elucidated [46].

**Figure 3:**
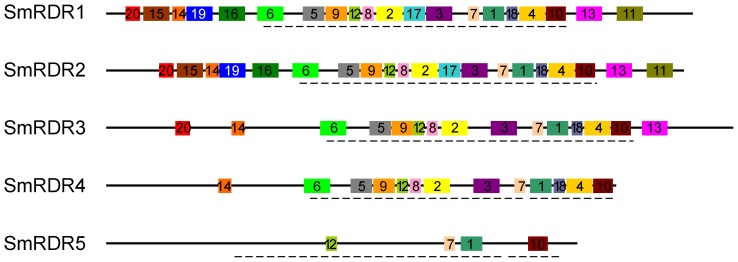
Conserved motifs of SmRDRs proteins identified with the MEME search tool. Motifs are represented by boxes. The numbers (1–20) and different colors in boxes represent motif 1–motif 20, respectively. Box size indicates the length of motifs. Broken lines indicate locations of the RdRp domain.

**Figure 4:**
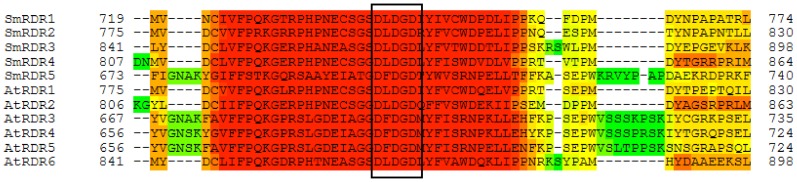
Alignment of partial sequences of RDR proteins in *S. miltiorrhiza* and *Arabidopsis* using T-coffee. The DLDGD/DFDGD signature is boxed. Different colors represent the quality of alignment with red for the highest quality, yellow for average, and green for the worst.

Using the MEME motif search tool, we analyzed conserved motifs of SmRDRs (Fig. 3). The results revealed four motifs (1, 7, 10 and 12) conserved in all of the five SmRDRs. It suggests the conservation of SmRDRs. On the other hand, various less conserved motifs were found. For instance, motifs 11, 15, 16, 17 and 19 are specific to SmRDR1 and SmRDR2. Motifs 13 and 20 are specific to SmRDR1, SmRDR2 and SmRDR3. Additionally, among the five SmRDRs, SmRDR1 and SmRDR2 have 20 motifs, whereas SmRDR5 only contain 4. These less conserved motifs could be associated with gene-specific functions. Sequence alignment of RDR proteins from *S. miltiorrhiza* and *Arabidopsis* using T-coffee [38] showed that SmRDRs contained the DLDGD/DFDGD signature, the partial sequence of motif 1 (Figs. 3 and 4). DLDGD/DFDGD has been previously found in various other plant RDRs and seems to be part of the nucleotidyl transferase active site of RDR proteins [47]. It further confirms the role of identified SmRDRs in the conversion of ssRNAs into dsRNAs.

### Phylogenetic analysis of RDR proteins in *S. miltiorrhiza*, *Arabidopsis* and rice

**Figure 5:**
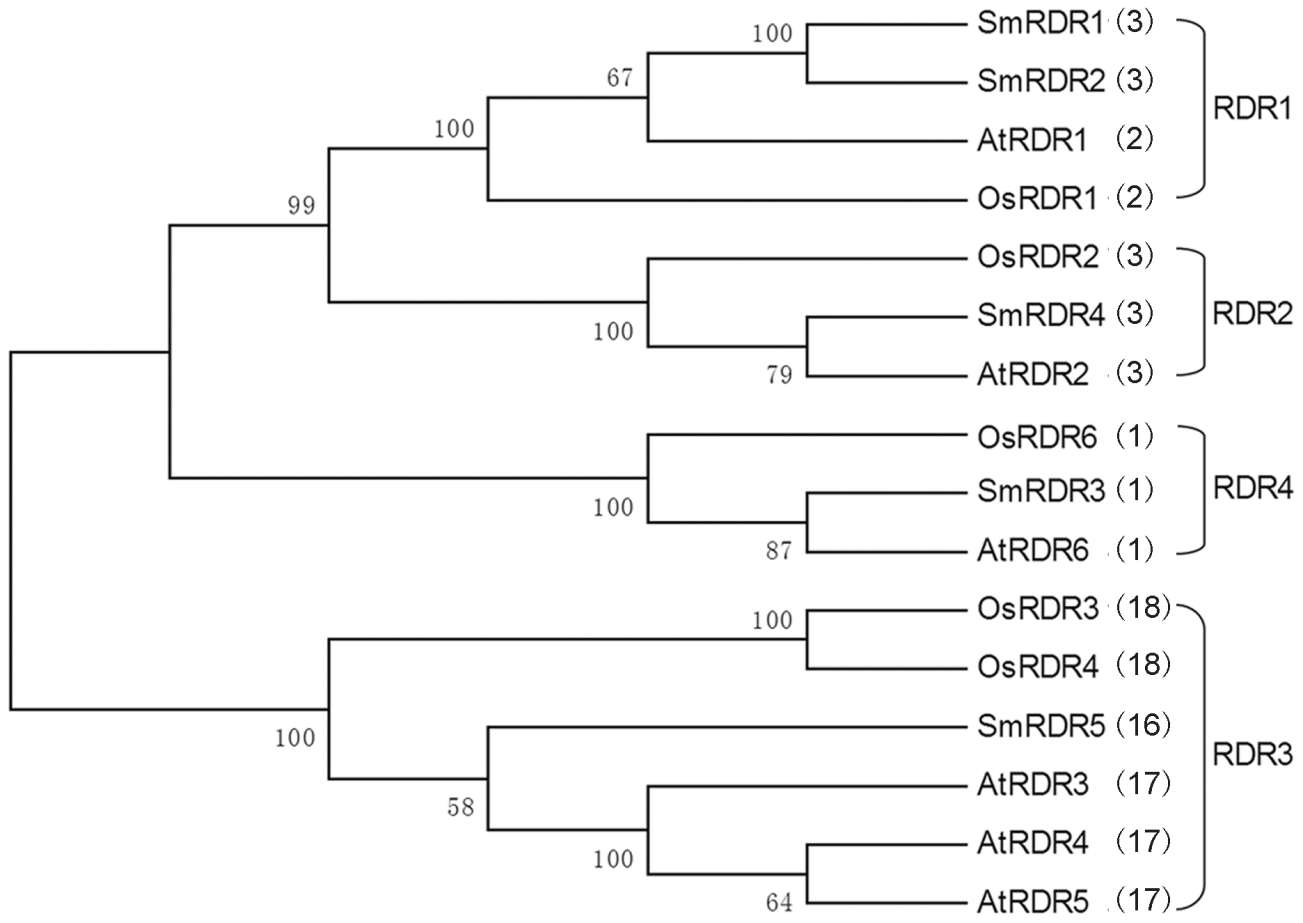
Phylogenetic relationships of sixteen RDRs from *S. miltiorrhiza*, *Arabidopsis* and rice. The relationships were analyzed for deduced full-length amino acid sequences using MEGA 4.0 by the neighbor-joining (NJ) method with 1000 bootstrap replicates. Bootstrap values are shown near the nodes. Four groups of RDRs, termed RDR1, RDR2, RDR3 and RDR4, respectively, are indicated. The number of introns in open reading frame (ORF) of the corresponding *RDR* gene is shown in parentheses.

Previous study revealed that RDRs in eukaryotic organisms might be divided into three clades, RDRα, RDRβ, and RDRγ [47]. RDRαproteins exist in all three kingdoms, whereas the proteins included in the RDRβ clades are present only in animals and fungi and RDRγproteins are found only in plants and fungi. Among the six *Arabidopsis* AtRDRs, AtRDR1, AtRDR2 and AtRDR6 belong to the RDRα clade, while AtRDR3, AtRDR4 and AtRDR5 are included in the RDRγclade [47]. To determine the evolutionary relationship among RDRs from *S. miltiorrhiza*, *Arabidopsis* and rice, an unrooted neighbor-joining tree was constructed for the full-length protein sequences of five SmRDRs, six AtRDRs and five OsRDRs. The results showed that the sixteen RDRs might be divided into four groups, termed RDR1, RDR2, RDR3 and RDR4, respectively (Fig. 5). It is consistent with previous results for RDRs from *Arabidopsis*, rice and *Zea mays* [15]–[17]. The RDR1, RDR2 and RDR4 groups contain SmRDR1, SmRDR2, SmRDR3 and SmRDR4 from *S. miltiorrhiz*a, AtRDR1, AtRDR2 and AtRDR6 from *Arabidopsis*, and OsRDR1, OsRDR2 and OsRDR6 from rice. All of them belong to the RDRαclade and are characterized by the DLDGD signature (Fig. 4). SmRDR1 and SmRDR2 and the antiviral defense-associated AtRDR1 and OsRDR1 [15], [16] are included in the RDR1 group. Members of the RDR2 group includes SmRDR4, *Arabidopsis* AtRDR2 and rice OsRDR2, of which AtRDR2 and OsRDR2 are involved in the production of the most abundant endogenous hsiRNAs and are responsible for siRNA-mediated DNA methylation and histone modifications at telomeres [25]. The RDR4 group contains SmRDR3, AtRDR6 and OsRDR6. AtRDR6 is associated with amplifying improper terminated and unpolyadenylated RNAs generated from transgenes or inverted repeats [27]. The RDR3 group includes AtRDR3, AtRDR4 and AtRDR5 from *Arabidopsis*, OsRDR3 and OsRDR4 from rice and SmRDR5 from *S. miltiorrhiza*. Members of the RDR3 group belong to the RDRγclade and are characterized by the DFDGD signature (Fig. 4). Although it is the biggest group, the function of RDRs in the RDR3 group is currently unknown.

### Tissue-specific expression of *SmRDR* genes

**Figure 6:**
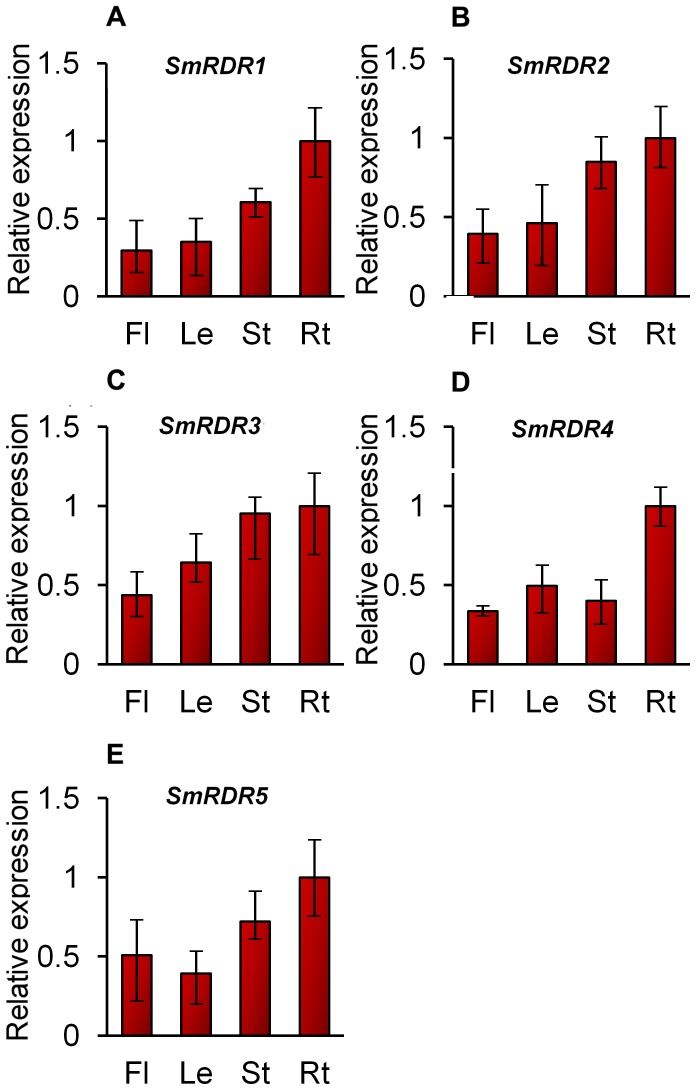
Expression of *SmRDRs* in flowers (Fl), leaves (Le), stems (St) and roots (Rt) of *S. miltiorrhiza*. The expression patterns were analyzed using the quantitative RT-PCR method. PCR was carried out in triplicates for each biological sample. Three independent biological replicates were performed. *SmUBQ10* was used as a reference. Fold changes of *SmRDR* expression are shown. The levels in roots were arbitrarily set to 1 and the levels in other tissues were given relative to this. Error bars represent the standard deviations of the mean value of three biological replicates.

To preliminarily elucidate the function of *SmRDR* genes, we analyzed the expression patterns of five identified *SmRDRs* in flowers, leaves, stems and roots of 2-year-old and field nursery-grown *S. miltiorrhiza* using the quantitative RT-PCR technology. The transcripts of all five *SmRDRs* could be detected in the tissues analyzed (Fig. 6), which is consistent with the vital roles of *RDRs* in plants. All of them showed the highest expression in roots and less in flowers and leaves (Fig.6). Further sequencing and analyzing the sRNAome in *S. miltiorrhiza* may help to elucidate the underlying mechanisms.

### The response of *SmRDRs* to MeJA treatment and CMV infection

**Figure 7:**
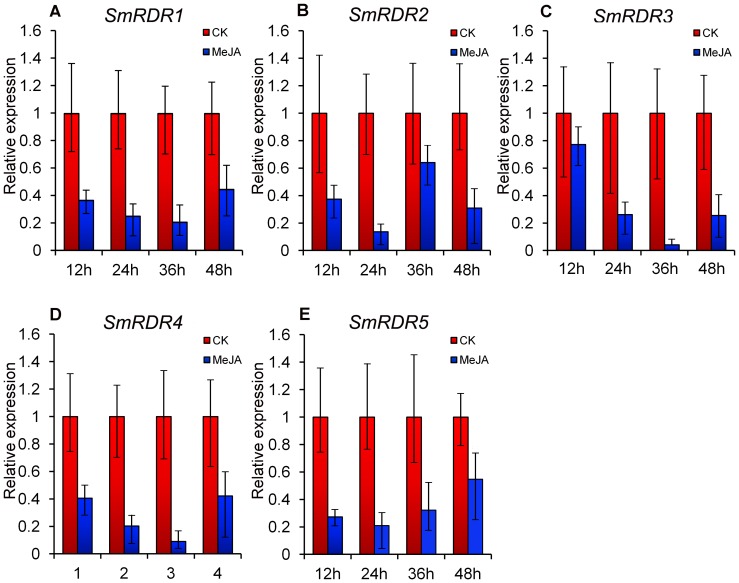
*SmRDRs* responsive to MeJA treatment. The expression patterns were analyzed using the quantitative RT-PCR method. PCR was carried out in triplicates for each biological sample. Three independent biological replicates were performed. *SmUBQ10* was used as a reference. Fold changes of *SmRDRs* in leaves of *S. miltiorrhiza* plantlets treated with MeJA for 12, 24, 36 and 48 h are shown. The level of transcripts in leaves treated with sterile water (CK) was arbitrarily set to 1 and the level in tissues treated with MeJA was given relative to this. Error bars represent standard deviations of mean value from three biological replicates.

RDRs catalyze the conversion of single-stranded RNAs into double-stranded ones and are core components for the production of siRNAs involved in plant development and response to abiotic and biotic stresses. In order to investigate the expression pattern of *SmRDR* genes under abiotic treatments, the expression level of *SmRDRs* in leaves of plantlets treated with MeJA was analyzed using the quantitative RT-PCR method. Plantlets treated with sterile water were used as controls. The results showed that all five *SmRDRs* were suppressed under MeJA treatment (Fig. 7). MeJA usually up-regulates the expression of genes associated with the biosynthesis of secondary metabolites, which play significant roles in plant response to stress [32], [33]. Down-regulation of *SmRDR* gene expression could be helpful to increase the production of stress-related secondary metabolites.

**Figure 8:**
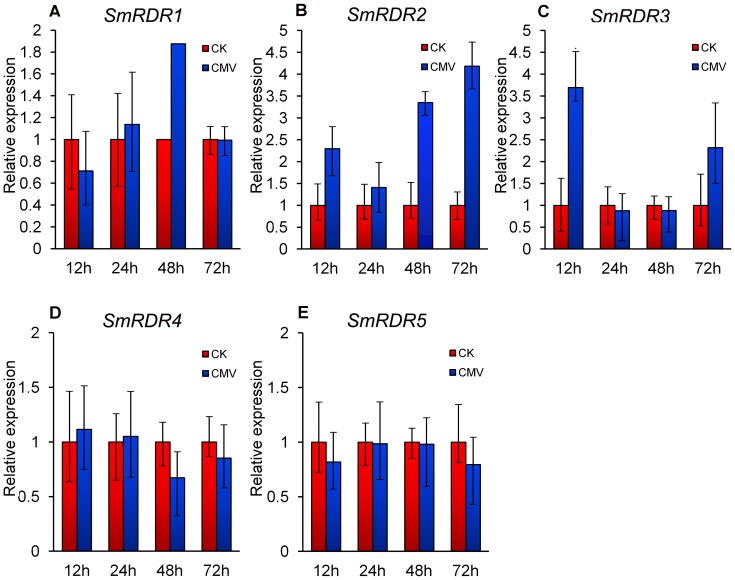
*SmRDRs* responsive to CMV infection. The expression patterns were analyzed using the quantitative RT-PCR method. PCR was carried out in triplicates for each biological sample. Three independent biological replicates were performed. *SmUBQ10* was used as a reference. Fold changes of *SmRDRs* in leaves of *S. miltiorrhiza* plantlets infected with CMV for 12, 24, 48 and 72 h are shown. The level of transcripts in leaves inoculated with phosphate buffered saline (CK) was arbitrarily set to 1 and the level in tissues inoculated with CMV was given relative to this. Error bars represent standard deviations of mean value from three biological replicates.

To examine the response of *SmRDRs* in biotic stress, *S. miltiorrhiza* plantlets were inoculated with CMV using the silicon carbide powder friction method. The level of *SmRDR* transcripts in leaves treated for 12, 24, 48 and 72 h was analyzed using the quantitative RT-PCR method. Leaves inoculated with phosphate buffered saline (PBS) were used as controls. As shown in Fig. 8, *SmRDR1*, *SmRDR2* and *SmRDR3* were up-regulated at different time points. The level of *SmRDR1* showed a 1.9-fold-increase after CMV inoculation for 48 h (Fig. 8A). *SmRDR2* showed 2.3-, 3.4- and 4.2-fold-increase after being treated with CMV for 12, 48 and 72 h, respectively (Fig. 8B). *SmRDR3* was accumulated to 3.7- and 2.3-folds of controls after CMV infection for 12 and 72 h (Fig. 8C). However, no significant change was observed for the level of *SmRDR4* and *SmRDR5* after CMV infection (Figs. 8D and 8E). The results indicate that *SmRDR1*, *SmRDR2* and *SmRDR3* may be involved in antiviral defense in *S. miltiorrhiza*.

## Discussion

*S. miltiorrhiza* is an economically significant medicinal plant species belonging to the largest genus, *Salvia*, in the mint family. It is native to China and Japan and is widely distributed in China. *S. miltiorrhiza* has close phylogenetic relationships with other Asian and Mediterranean *Salvia* species, such as *S. roborowskii* and *S. glutinosa* [48]. The root of *S. miltiorrhiza* has been widely used in TCMs for hundred of years to treat dysmenorrhoea, amenorrhoea and cardiovascular diseases [28]–[30]. The main bioactive components in *S. miltiorrhiza* are lipophilic diterpenoid tanshinones and hydrophilic phenolic acids. Genes involved in the biosynthesis of these components have been intensely studied recently [32], [33], [49]–[51]. Because of its relatively small genome size, short life cycle, undemanding growth requirements and significant medicinal value, *S. miltiorrhiza* is being developed to become a model medicinal plant for TCM studies [31]–[33]. Elucidation of small RNA biogenesis pathways in *S. miltiorrhiza* appears to be urgent, given the significant regulatory roles of small RNAs in plant development and growth. Results from *Arabidopsis* suggest that the core components of small RNA pathways include at least three gene families, *DCL*, *AGO* and *RDR* [2]. The *AGO* gene family in *S. miltiorrhiza* has been previously characterized by our research group [31].

RDRs are core components of various gene silencing pathways and play vital roles in plant development and antiviral defense through regulating gene expression at the transcriptional and post-transcriptional levels [52]. *RDR* genes have been identified in various plants, such as *Arabidopsis* [14], rice [16] and maize [17]. However, many of them were computationally predicted only. For instance, among the six *Arabidopsis AtRDRs*, only three, including *AtRDR1*, AtRDR2 and *AtRDR6*, were cloned [7]. Of the five rice *OsRDRs*, only Os*RDR6* were experimentally isolated [53]. Through genome-wide analysis of the working draft of the *S. miltiorrhiza* genome, we identified a total of five *SmRDRs* in *S. miltiorrhiza*. The full-length cDNAs of all predicted *SmRDRs* were then cloned and characterized. To our best knowledge, it is the first set of full-length *SmRDR* cDNAs from *S. miltiorrhiza*. The results provide useful information for further elucidation of *RDR* functions in *S. miltiorrhiza*.

Using a comprehensive approach, which combines sequence feature, gene structure, conserved domain and phylogenetic analyses, we characterized the five identified *SmRDRs*. The genomic sequence of *SmRDR3* generating the longest *SmRDR* cDNA contains three introns, all of which locate in the open reading frame (ORF); whereas the *SmRDR5* gene that produces the shortest *SmRDR* cDNA has seventeen introns with sixteen in ORF, suggesting the wide range of intron number in *SmRDRs*. The results are consistent with those from *Arabidopsis* and rice. The numbers of introns in *AtRDR* and *OsRDR* ORFs vary from 1 (*AtRDR6*) to 17 (*AtRDR3*, *AtRDR4* and *AtRDR5*) and 1 (*OsRDR6*) to 18 (*OsRDR4*), respectively [16]. Examination of intron numbers in the ORF of *S. miltiorrhiza*, *Arabidopsis* and rice *RDR* genes suggest that *RDRs* in each phylogenetic group have similar number of introns (Fig. 1) [16]. For instance, all RDR2 group members, including *SmRDR4*, *AtRDR2* and *OsRDR2*, have 3 introns. The members in the RDR3 group contain 16–18 introns. It suggests the close evolutionary relationship of *RDRs* in a phylogenetic group.

Phylogenetic analysis of RDR proteins in *S. miltiorrhiza*, *Arabidopsis* and rice showed that RDRs clustered into four distinct groups (Fig. 5). Members of the RDR1, RDR2 and RDR6 groups are RDRα-type proteins characterized by the DLDGD signature (Fig. 4). There is only one RDRα-type RDR in the most recent common ancestor of plants, animals and fungi [47]. However, there are four in *S. miltiorrhiza*, of which, two are in the RDR1 group, one belongs in the RDR2 group, and the other one is included in the RDR4 group (Fig. 5). It suggests the occurrence of gene duplication for the RDRα-type gene during *S. miltiorrhiza* evolution. Since these RDRα-type RDRs clustered into different groups, they could be functionally diversified. All of the RDRγ-type RDRs, which are characterized by the DFDGD signature (Fig. 4), clustered in the RDR3 group (Fig. 5). It includes three *Arabidopsis* RDRs (AtRDR3–AtRDR5), two rice RDRs (OsRDR3 and OsRDR4); however, there is only one *S. miltiorrhiza* RDRs (SmRDR5). It suggests that gene duplication has occurred for the RDRγ-type gene during *Arabidopsis* and rice evolution but not in *S. miltiorrhiza* evolution.

Gene expression analysis showed that *SmRDRs* were expressed in all of the tissues detected and responsive to MeJA treatment and CMV infection. All five *SmRDRs* were suppressed under MeJA treatment (Fig. 7), whereas *SmRDR1*, *SmRDR2* and *SmRDR3* were up-regulated after CMV inoculation (Fig. 8). The involvement of *S. miltiorrhiza SmRDRs* in anti-viral defense is consistent with previous results for *Arabidopsis AtRDR1*, an ortholog of *SmRDR1* and *SmRDR2*, and *AtRDR6*, an ortholog of *SmRDR3* [7]. Further genetic manipulation of *SmRDRs* will shed light on the small RNA biogenesis pathways and *RDR* functions in *S. miltiorrhiza*.

## Supporting Information

Table S1 **Primers used for 5′-RACE of** ***SmRDRs*** **.**
(DOC)

Table S2 **Primers used for 3′-RACE of** ***SmRDRs*** **.**
(DOC)

Table S3 **Primers used for amplification of full-length** ***SmRDR*** **cDNAs.**
(DOC)

Table S4 **Primers used for qRT-PCR.**
(DOC)

## References

[1] Moissiard G, Parizotto EA, Himber C, Voinnet O (2007) Transitivity in *Arabidopsis* can be primed, requires the redundant action of the antiviral Dicer-like 4 and Dicer-like 2, and is compromised by viral-encoded suppressor proteins. RNA 13: 1268–1278. 17592042 10.1261/rna.541307 PMC1924903

[2] Voinnet O (2009) Origin, biogenesis, and activity of plant microRNAs. Cell 136: 669–687. 19239888 10.1016/j.cell.2009.01.046

[3] Bartel DP (2004) MicroRNAs: genomics, biogenesis, mechanism, and function. Cell 116: 281–297. 14744438 10.1016/s0092-8674(04)00045-5

[4] Baulcombe D (2004) RNA silencing in plants. Nature 431: 356–363. 15372043 10.1038/nature02874

[5] Carthew RW, Sontheimer EJ (2009) Origins and mechanisms of miRNAs and siRNAs. Cell 136: 642–655. 19239886 10.1016/j.cell.2009.01.035 PMC2675692

[6] Johnston M, Hutvagner G (2011) Posttranslational modification of Argonautes and their role in small RNA-mediated gene regulation. Silence 2: 5. 21943311 10.1186/1758-907X-2-5 PMC3199228

[7] Willmann MR, Endres MW, Cook RT, Gregory BD (2011) The functions of RNA-dependent RNA polymerases in *Arabidopsis* . The Arabidopsis Book 9: e0146. 22303271 10.1199/tab.0146 PMC3268507

[8] Astier-Manifacier S, Cornuet P (1971) RNA-dependent RNA polymerase in Chinese cabbage. Biochim Biophys Acta 232: 484–493. 5572618 10.1016/0005-2787(71)90602-2

[9] Dalmay T, Hamilton A, Rudd S, Angell S, Baulcombe DC (2000) An RNA-dependent RNA polymerase gene in *Arabidopsis* is required for posttranscriptional gene silencing mediated by a transgene but not by a virus. Cell 101: 543–553. 10850496 10.1016/s0092-8674(00)80864-8

[10] Mourrain P, Beclin C, Elmayan T, Feuerbach F, Godon C, et al (2000) *Arabidopsis* SGS2 and SGS3 genes are required for posttranscriptional gene silencing and natural virus resistance. Cell 101: 533–542. 10850495 10.1016/s0092-8674(00)80863-6

[11] Cogoni C, Macino G (1999) Homology-dependent gene silencing in plants and fungi: a number of variations on the same theme. Curr Opin Microbiol 2: 657–662. 10607623 10.1016/s1369-5274(99)00041-7

[12] Djupedal I, Ekwall K (2009) Epigenetics: heterochromatin meets RNAi. Cell Res 19: 282–295. 19188930 10.1038/cr.2009.13

[13] Schiebel W, Pélissier T, Riedel L, Thalmeir S, Schiebel R, et al (1998) Isolation of an RNA-directed RNA polymerase-specific cDNA clone from tomato. Plant Cell 10: 2087–2101. 9836747 10.1105/tpc.10.12.2087 PMC143969

[14] Wassenegger M, Krczal G (2006) Nomenclature and functions of RNA-directed RNA polymerases. Trends Plant Sci 11: 142–151. 16473542 10.1016/j.tplants.2006.01.003

[15] Bai M, Yang GS, Chen WT, Mao ZC, Kang HX, et al (2012) Genome-wide identification of Dicer-like, Argonaute and RNA-dependent RNA polymerase gene families and their expression analyses in response to viral infection and abiotic stresses in *Solanum lycopersicum* . Gene 501: 52–62. 22406496 10.1016/j.gene.2012.02.009

[16] Kapoor M, Arora R, Lama T, Nijhawan A, Khurana JP, et al (2008) Genome-wide identification, organization and phylogenetic analysis of *Dicer*-like, Argonaute and RNA-dependent RNA polymerase gene families and their expression analysis during reproductive development and stress in rice. BMC Genomics 9: 451. 18826656 10.1186/1471-2164-9-451 PMC2576257

[17] Qian Y, Cheng Y, Cheng X, Jiang H, Zhu S, et al (2011) Identification and characterization of Dicer-like, Argonaute and RNA-dependent RNA polymerase gene families in maize. Plant Cell Rep 30: 1347–1363. 21404010 10.1007/s00299-011-1046-6

[18] Pandey SP, Baldwin IT (2007) RNA-directed RNA polymerase1 (RdR1) mediates the resistance of *Nicotiana attenuata* to herbivore attack in nature. Plant J 50: 40–53. 17346266 10.1111/j.1365-313X.2007.03030.x

[19] Diaz-Pendon JA, Li F, Li WX, Ding SW (2007) Suppression of antiviral silencing by cucumber mosaic virus 2b protein in *Arabidopsis* is associated with drastically reduced accumulation of three classes of viral small interfering RNAs. Plant Cell 19: 2053–2063. 17586651 10.1105/tpc.106.047449 PMC1955711

[20] Donaire L, Barajas D, Martinez-Garcia B, Martinez-Priego L, Pagan I, et al (2008) Structural and genetic requirements for the biogenesis of tobacco rattle virus-derived small interfering RNAs. J Virol 82: 5167–5177. 18353962 10.1128/JVI.00272-08 PMC2395200

[21] Yu D, Fan B, MacFarlane SA, Chen Z (2003) Analysis of the involvement of an inducible Arabidopsis RNA-dependent RNA polymerase in antiviral defense. Mol Plant Microbe Interact 16: 206–216. 12650452 10.1094/MPMI.2003.16.3.206

[22] Qi X, Bao FS, Xie Z (2009) Small RNA deep sequencing reveals role for *Arabidopsis thaliana* RNA-dependent RNA polymerases in viral siRNA biogenesis. PLoS One 4: e4971. 19308254 10.1371/journal.pone.0004971 PMC2654919

[23] Wang XB, Wu Q, Ito T, Cillo F, Li WX, et al (2010) RNAi-mediated viral immunity requires amplification of virus-derived siRNAs in *Arabidopsis thaliana* . Proc Natl Acad Sci USA 107: 484–489. 19966292 10.1073/pnas.0904086107 PMC2806737

[24] Qu F, Ye X, Morris TJ (2008) *Arabidopsis* DRB4, AGO1, AGO7, and RDR6 participate in a DCL4-initiated antiviral RNA silencing pathway negatively regulated by DCL1. Proc Natl Acad Sci USA 105: 14732–14737. 18799732 10.1073/pnas.0805760105 PMC2567185

[25] Vrbsky J, Akimcheva S, Watson JM, Turner TL, Daxinger L, et al (2010) siRNA-mediated methylation of *Arabidopsis* telomeres. PLoS Genet 6: e1000986. 20548962 10.1371/journal.pgen.1000986 PMC2883606

[26] Olmedo-Monfil V, Duran-Figueroa N, Arteaga-Vazquez M, Demesa-Arevalo E, Autran D, et al (2010) Control of female gamete formation by a small RNA pathway in *Arabidopsis* . Nature 464: 628–632. 20208518 10.1038/nature08828 PMC4613780

[27] Luo Z, Chen Z (2007) Improperly terminated, unpolyadenylated mRNA of sense transgenes is targeted by RDR6-mediated RNA silencing in *Arabidopsis* . Plant Cell 19: 943–958. 17384170 10.1105/tpc.106.045724 PMC1867362

[28] Cheng TO (2006) Danshen a popular chinese cardiac herbal drug. J Am Coll Cardiol 47: 1498. 10.1016/j.jacc.2006.01.001 16580549

[29] He S, Yang Y, Liu X, Huang W, Zhang X, et al (2012) Compound astragalus and Salvia miltiorrhiza extract inhibits cell proliferation, invasion and collagen synthesis in keloid fibroblasts by mediating TGF-beta/smad pathway. Br J Dermatol 166: 564–574. 21967214 10.1111/j.1365-2133.2011.10674.x

[30] Wen JB, Wu JY (2010) Tanshinone biosynthesis in Salvia miltiorrhiza and production in plant tissue cultures. Appl Microbiol Biotechnol 88: 437–449. 20694462 10.1007/s00253-010-2797-7

[31] Shao F, Lu S (2013) Genome-wide identification, molecular cloning, expression profiling and posttranscriptional regulation analysis of the Argonaute gene family in *Salvia miltiorrhiza*, an emerging model medicinal plant. BMC Genomics 14: 512. 23889895 10.1186/1471-2164-14-512 PMC3750313

[32] Ma Y, Yuan L, Wu B, Li X, Chen S, et al (2012) Genome-wide identification and characterization of novel genes involved in terpenoid biosynthesis in *Salvia miltiorrhiza* . J Exp Bot 63: 2809–2823. 22291132 10.1093/jxb/err466 PMC3346237

[33] Hou X, Shao F, Ma Y, Lu S (2013) The phenylalanine ammonia-lyase gene family in *Salvia miltiorrhiza*: genome-wide characterization, molecular cloning and expression analysis. Mol Biol Rep 40: 4301–4310. 23644983 10.1007/s11033-013-2517-3

[34] Altschul S, Madden T, Schaffer A, Zhang J, Zhang Z, et al (1997) Gapped BLAST and PSI-BLAST: a new generation of protein database search programs. Nucleic Acids Res 25: 3389–3402. 9254694 10.1093/nar/25.17.3389 PMC146917

[35] Burge C, Karlin S (1998) Finding the genes in genomic DNA. Curr Opin Struct Biol 8: 346–354. 9666331 10.1016/s0959-440x(98)80069-9

[36] Marchler-Bauer A, Lu S, Anderson JB, Chitsaz F, Derbyshire MK, et al (2010) CDD: a conserved domain database for the functional annotation of proteins. Nucleic Acids Res 39: D225–D229. 21109532 10.1093/nar/gkq1189 PMC3013737

[37] Bailey TL, Elkan C (1994) Fitting a mixture model by expectation maximization to discover motifs in biopolymers. Proc Int Conf Intell Syst Mol Biol 2: 28–36. 7584402

[38] Notredame C, Higgins DG, Heringa J (2000) T-Coffee: A novel method for fast and accurate multiple sequence alignment. J Mol Biol 302: 205–17. 10964570 10.1006/jmbi.2000.4042

[39] Thompson J, Higgins D, Gibson T (1994) CLUSTAL W: improving the sensitivity of progressive multiple sequence alignment through sequence weighting, position-specific gap penalties and weight matrix choice. Nucleic Acids Res 22: 4673–4680. 7984417 10.1093/nar/22.22.4673 PMC308517

[40] Kumar S, Tamura K, Nei M (2004) MEGA3 integrated software for molecular evolutionary genetics analysis and sequence alignment. Brief Bioinform 5: 150–163. 15260895 10.1093/bib/5.2.150

[41] Livak KJ, Schmittgen TD (2001) Analysis of relative gene expression data using real-time quantitative PCR and the 2 (-Delta Delta C(T)) method. Methods 25: 402–408. 11846609 10.1006/meth.2001.1262

[42] Willems E, Leyns L, Vandesompele J (2008) Standardization of real-time PCR gene expression data from independent biological replicates. Anal Biochem 379: 127–129. 18485881 10.1016/j.ab.2008.04.036

[43] Lu J, Sivamani E, Azhakanandam K, Samadder P, Li X, et al (2008) Gene expression enhancement mediated by the 50 UTR intron of the rice rubi3 gene varied remarkably among tissues in transgenic rice plants. Mol Genet Genomics 279: 563–572. 18320227 10.1007/s00438-008-0333-6

[44] Bartlett JG, Snape JW, Harwood WA (2009) Intron-mediated enhancement as a method for increasing transgene expression levels in barley. Plant Biotechnol J 7: 856–866. 19781005 10.1111/j.1467-7652.2009.00448.x

[45] Parra G, Bradnam K, Rose AB, Korf I (2011) Comparative and functional analysis of intron-mediated enhancement signals reveals conserved features among plants. Nucleic Acids Res 39: 5328–5337. 21427088 10.1093/nar/gkr043 PMC3141229

[46] Birney E, Kumar S, Krainer AR (1993) Analysis of the RNA-recognition motif and RS and RGG domains: conservation in metazoan pre-mRNA splicing factors. Nucleic Acids Res 21: 5803–5816. 8290338 10.1093/nar/21.25.5803 PMC310458

[47] Zong J, Yao X, Yin J, Zhang D, Ma H (2009) Evolution of the RNA-dependent RNA polymerase (RdRP) genes: duplications and possible losses before and after the divergence of major eukaryotic groups. Gene 447: 29–39. 19616606 10.1016/j.gene.2009.07.004

[48] Walker JB, Sytsma KJ (2007) Staminal evolution in the genus *Salvia* (Lamiaceae): Molecular phylogenetic evidence for multiple origins of the staminal lever. Ann Bot 100: 375–391. 16926227 10.1093/aob/mcl176 PMC2735309

[49] Xiao Y, Zhang L, Gao S, Saechao S, Di P, et al (2011) The *c4h*, *tat*, *hppr* and *hppd* genes prompted engineering of rosmarinic acid biosynthetic pathway in *Salvia miltiorrhiza* hairy root cultures. PLoS One 6: e29713. 22242141 10.1371/journal.pone.0029713 PMC3248448

[50] Zhang L, Wu B, Zhao D, Li C, Shao F, et al (2014) Genome-wide analysis and molecular dissection of the *SPL* gene family in *Salvia miltiorrhiza* . J Integr Plant Biol 56: 38–50. 24112769 10.1111/jipb.12111

[51] Guo J, Zhou YJ, Hillwig ML, Shen Y, Yang L, et al (2013) CYP76AH1 catalyzes turnover of miltiradiene in tanshinones biosynthesis and enables heterologous production of ferruginol in yeasts. Proc Natl Acad Sci U S A 110: 12108–12113. 23812755 10.1073/pnas.1218061110 PMC3718111

[52] Carrington J (2003) Role of microRNAs in plant and animal development. Science 301: 336–338. 12869753 10.1126/science.1085242

[53] Nagasaki H, Itoh J, Hayashi K, Hibara K, Satoh-Nagasawa N, et al (2007) The small interfering RNA production pathway is required for shoot meristem initiation in rice. Proc Natl Acad Sci U S A 104: 14867–14871. 17804793 10.1073/pnas.0704339104 PMC1976227

