# Supplementary material for: Identification, Molecular Cloning and Expression Analysis of Five RNA-Dependent RNA Polymerase Genes in Salvia miltiorrhiza
Source: PLoS One. 2014 Apr 14;9(4):e95117. doi: 10.1371/journal.pone.0095117 (PMC3986363; doi:10.1371/journal.pone.0095117)
Supplement: Table S1 — Primers used for 5′-RACE of SmRDRs . (DOC) [file pone.0095117.s001.doc]

**Table S1. Primers used for 5’-RACE of *SmRDRs*.**

| **Gene name** | **Primer sequence (5' to 3')** |
| --- | --- |
| *SmRDR1* | Nesting: CAGAGTTACACCCTCCACAACAT |
|  | Nested: CTGCGTAAGTATGGCTGCTCCAA |
| *SmRDR2* | Nesting: CTTGTCGGCGTCTTCTTTAGAGGT |
|  | Nested: CTCCCTCGCACGTGTGACCCTCCA |
| *SmRDR3* | Nesting: GCCGAAGCTGATGACTGTGCAACT |
|  | Nested: GTCTTCACTCGACACCTCCAAACA |
| *SmRDR4* | Nesting: GCTCTCGAATTGGACGCGGCCAT |
|  | Nested: GGCTATGGCTATCGGTGGAATGT |
| *SmRDR5* | Nesting: GAGACGAAGCCACCGAAGCTT |
|  | Nested: GGAGTCTTGGAGGAGTGAATGAT |
