# Supplementary material for: Identification, Molecular Cloning and Expression Analysis of Five RNA-Dependent RNA Polymerase Genes in Salvia miltiorrhiza
Source: PLoS One. 2014 Apr 14;9(4):e95117. doi: 10.1371/journal.pone.0095117 (PMC3986363; doi:10.1371/journal.pone.0095117)
Supplement: Table S2 — Primers used for 3′-RACE of SmRDRs . (DOC) [file pone.0095117.s002.doc]

**Table S2. Primers used for 3’-RACE of *SmRDRs*.**

| **Gene name** | **Primer sequence (5' to 3')** |
| --- | --- |
| *SmRDR1* | Nesting: GTGCTGCAGTGAAGTCTCTGAGGA |
|  | Nested: GGCTTCGGCTTGGTATCACGTGA |
| *SmRDR2* | Nesting: CGCCAGATCATCACACTCTTGTCT |
|  | Nested: GTCGATCCACTCAAAGCTCAAGA |
| *SmRDR3* | Nesting: GACCAGCTCCCGGATCATGAGAA |
|  | Nested: GCTGGATGATTCTGGGAAGACAGT |
| *SmRDR4* | Nesting: GGACGACGGCCACGCATCATGAA |
|  | Nested: CGACACTCTGGGCGCGATCTCTA |
| *SmRDR5* | Nesting: GGATCGGCTGCTGACTCTAGGTGA |
|  | Nested: GGGAAACAGATCAATGTGCCTGA |

**Table S3. Primers used for amplification of full-length *SmRDR* cDNA*s*.**

| **Gene name** | **Primer Sequence (5' to 3')** |
| --- | --- |
| SmRDR1 | Forward: CTGACATTGTGCCAAATCCTAGAA |
|  | Reverse: CAGAGACTTCACTGCAGCACCAAT |
| *SmRDR2* | Forward: GCATGAATTGGATGGCCTCACTTT |
|  | Reverse: GAATCAACTCAGGGTCCCAACACA |
| *SmRDR3* | Forward: CGCAGTTGCACAGTCATCAGCTT |
|  | Reverse: CCGAGCAAGATAGTCAGCAGCTA |
| *SmRDR4* | Forward: GTCGAAATCTTCACGGAGCGTGA |
|  | Reverse: CGAGTAGTTCCCGTGGTACGTCA |
| *SmRDR5* | Forward: GAGTCCGTAGAAGCGGCCATCGAA |
|  | Reverse: CGGCATCGTTCTTGACGTCACCAA |

**Table S4. Primers used for qRT-PCR.**

| **Gene name** | **Primer Sequence (5' to 3')** |
| --- | --- |
| *SmUBQ10* | Forward: AGATGGGCGGACACTTGCTGATTA |
|  | Reverse: ACTCTCCACCTCCAAAGTGATGGT |
| *SmRDR1* | Forward: CCTGATGGACTACTACGGCATCA |
|  | Reverse: GATACCAAGCCGAAGCCATTGCA |
| *SmRDR2* | Forward: CTGTGCTGTGGAGGGCTGCAGAT |
|  | Reverse: CGAGGTGCTCCGAATAGCTGTAT |
| *SmRDR3* | Forward: GGACTTGTTTGGAGGTGTCGAGT |
|  | Reverse: GACAAGGTCTCCAGCACCTGATA |
| *SmRDR4* | Forward: GATCTTGTCCCACCTCGAACTGT |
|  | Reverse: GTTAGAGATCGCGCCCAGAGTGT |
| *SmRDR5* | Forward: GGGAAACAGATCAATGTGCCTGA |
|  | Reverse: CCTTTTCTCGACACAGCATCACT |
