# Supplementary material for: Identification, Molecular Cloning and Expression Analysis of Five RNA-Dependent RNA Polymerase Genes in Salvia miltiorrhiza
Source: PLoS One. 2014 Apr 14;9(4):e95117. doi: 10.1371/journal.pone.0095117 (PMC3986363; doi:10.1371/journal.pone.0095117)
Supplement: Table S3 — Primers used for amplification of full-length SmRDR cDNAs. (DOC) [file pone.0095117.s003.doc]

**Table S3. Primers used for amplification of full-length *SmRDR* cDNAs.**

| **Gene name** | **Primer sequence (5' to 3')** |
| --- | --- |
| *SmRDR1* | Forward: CTGACATTGTGCCAAATCCTAGAA |
|  | Reverse: CAGAGACTTCACTGCAGCACCAAT |
| *SmRDR2* | Forward: GCATGAATTGGATGGCCTCACTTT |
|  | Reverse: GAATCAACTCAGGGTCCCAACACA |
| *SmRDR3* | Forward: CGCAGTTGCACAGTCATCAGCTT |
|  | Reverse: CCGAGCAAGATAGTCAGCAGCTA |
| *SmRDR4* | Forward: GTCGAAATCTTCACGGAGCGTGA |
|  | Reverse: CGAGTAGTTCCCGTGGTACGTCA |
| *SmRDR5* | Forward: GAGTCCGTAGAAGCGGCCATCGAA |
|  | Reverse: CGGCATCGTTCTTGACGTCACCAA |
