# Supplementary material for: Identification, Molecular Cloning and Expression Analysis of Five RNA-Dependent RNA Polymerase Genes in Salvia miltiorrhiza
Source: PLoS One. 2014 Apr 14;9(4):e95117. doi: 10.1371/journal.pone.0095117 (PMC3986363; doi:10.1371/journal.pone.0095117)
Supplement: Table S4 — Primers used for qRT-PCR. (DOC) [file pone.0095117.s004.doc]

**Table S4. Primers used for qRT-PCR.**

| **Gene name** | **Primer sequence (5' to 3')** |
| --- | --- |
| *SmUBQ10* | Forward: AGATGGGCGGACACTTGCTGATTA |
|  | Reverse: ACTCTCCACCTCCAAAGTGATGGT |
| *SmRDR1* | Forward: CCTGATGGACTACTACGGCATCA |
|  | Reverse: GATACCAAGCCGAAGCCATTGCA |
| *SmRDR2* | Forward: CTGTGCTGTGGAGGGCTGCAGAT |
|  | Reverse: CGAGGTGCTCCGAATAGCTGTAT |
| *SmRDR3* | Forward: GGACTTGTTTGGAGGTGTCGAGT |
|  | Reverse: GACAAGGTCTCCAGCACCTGATA |
| *SmRDR4* | Forward: GATCTTGTCCCACCTCGAACTGT |
|  | Reverse: GTTAGAGATCGCGCCCAGAGTGT |
| *SmRDR5* | Forward: GGGAAACAGATCAATGTGCCTGA |
|  | Reverse: CCTTTTCTCGACACAGCATCACT |
